# Caregivers’ Baseline Mental Health Problems and Early Childhood Social Skills at One-Year Follow-Up in an Urban Area of Indonesia

**DOI:** 10.3390/children13040508

**Published:** 2026-04-04

**Authors:** Hilda Meriyandah, Yuri Nurdiantami, Smarika Shresta, Maiko Shigeeda, Tokie Anme

**Affiliations:** 1 Graduate School of Comprehensive Human Sciences, University of Tsukuba, 1-1-1 Tennodai, Ibaraki, Tsukuba 305-8577, Japan; s2236041@u.tsukuba.ac.jp (H.M.); nurdiantamiyuri@upnvj.ac.id (Y.N.); smarikashrs@gmail.com (S.S.); maiko.shigeeda@gmail.com (M.S.); 2 Faculty of Health Sciences, Universitas Pembangunan Nasional Veteran Jakarta, Jl. RS Fatmawati, Pondok Labu, Jakarta 12450, Indonesia; 3 Faculty of Medicine, University of Tsukuba, 1-1-1 Tennodai, Ibaraki, Tsukuba 305-8575, Japan

**Keywords:** caregivers, follow-up approach, mental health, preschoolers, social development, urban

## Abstract

**Highlights:**

**What are the main findings?**

- Caregivers’ depression, anxiety, and stress problems at baseline were associated with lower social skill development after one year in an urban Indonesian context.
- The associations remained significant after adjusting for covariates and clustering at the kindergarten level by mixed modeling.

**What are the implications of the main findings?**

- Mental health screenings should be integrated by utilizing a valid checklist for caregivers for the early identification of at-risk families.
- Strengthening awareness and accessibility of caregiver mental health services may contribute to more comprehensive approaches to promoting child development.

**Abstract:**

Background/Objectives: Social development in children is a significant aspect that supports appropriate behavior in the community, and parents, as the main caregivers, play a central role in developing social skills in children. However, caregivers experiencing mental health problems—such as depression, anxiety, and stress—may find it challenging to provide a nurturing rearing environment. This one-year follow-up study examined whether the baseline mental health of caregivers was associated with social skills in children 1 year later in an urban Indonesian context. Methods: A one-year follow-up study was conducted in an urban area of Indonesia in 2023–2024, inviting all nine kindergartens in the area to participate. Caregivers completed the demographic questionnaire and the Depression, Anxiety, Stress Scale (DASS-21), while teachers assessed social skills in children using the Social Skills Scale (SSS). Linear mixed-effects models with random intercepts for kindergarten were estimated to account for clustering. Results: Finally, a total of 270 parent–child dyads were included. After adjusting for baseline social skills and covariates, higher levels of baseline caregiver depression (B = −0.15, *p* < 0.001), anxiety (B = −0.22, *p* < 0.001), and stress (B = −0.27, *p* < 0.001) were associated with lower social skills in children in the follow-up. Conclusions: Even subclinical variations in caregiver mental health problems may be meaningfully associated with social development in children over time. The findings highlight mental health in caregivers as a potentially important factor associated with early social development in an urban setting of Indonesia.

## 1. Introduction

Early childhood growth and development are major and extensively debated topics. They encompass more than just health and the capacity to engage in various activities; they entail a broader dimension, including social–emotional development [1]. Previous research highlights the importance of social skills as fundamental competencies that children must cultivate from an early age, as they are important in how children can adapt well to their environment. Advanced studies justified social skills as learned behaviors based on social rules enabling children to interact appropriately with others in society [2,3].

Several advanced studies have indicated that a deficiency in social skill development from early childhood is associated with adverse adult outcomes across various domains. These effects include an increased risk of unemployment, financial difficulties, a higher likelihood of developing obesity, and involvement in certain criminal activities [4]. Richards and Huppert propose that children deficient in essential social skills may encounter difficulties in adulthood due to the development of unfavorable temperaments [5]. Conversely, enhancements in social skills throughout the preschool years might result in success in later life, higher life satisfaction and well-being, better labor market performance, good health, and a healthy relationship status [4]. These findings emphasize that successful development in childhood plays an important role in later adult outcomes.

Recent studies have suggested an increase in the prevalence of young children exhibiting problematic behavior, marked by reduced social and emotional competencies [6,7]. A study in Indonesia also reported that many children demonstrate limited social skills. Some preschoolers exhibit a preference for solitude or shyness, struggle to regulate their emotions and behaviors, do not wish to share, and have difficulties collaborating with peers [8].

Numerous prior studies have examined the potential factors of social skills in children, highlighting the importance of both internal and external factors, such as age, gender, family environment, parent–child relationship, parenting style, and parental mental health [9,10,11,12]. All these factors surround the children and shape their social development.

The mental health of caregivers is considered a crucial aspect of preschooler development, particularly regarding social skills in children. Advanced studies indicate that parents with mental health illnesses have an elevated risk of having children who experience social developmental issues [13,14]. Particularly in low- and middle-income countries (LMICs), research has found that, when parents have mental health problems, their children under five are about twice as likely to have unfavorable social and emotional outcomes [14]. Another study also found that parental stress during the infancy period of life would increase the risk of 3-year-old children developing mental health problems [15], and a prior study in Indonesia indicated that preschool children reared by women under stress are 19.5 times more susceptible to encountering inadequate parenting and are at risk of developing mental problems [16]. This risk grows with the severity of the caregivers’ mental health problems. Particularly in LMIC settings, caregivers may experience additional stressors, including economic constraints, limited access to mental health services, and densely populated urban environments [17,18]. These contextual factors may amplify the association between caregiver mental health problems and developmental outcomes in children. However, evidence of follow-up studies from LMICs remains limited, particularly in Southeast Asian contexts.

In this study, we explain how mental health in caregivers is associated with social skills development in children by utilizing two theoretical frameworks: the Family Stress Model and Social Learning Theory. The former is introduced to elucidate how stressors can induce parental distress—manifesting as depression, anxiety, and stress—which may result in less responsive parenting practices. These alter parenting behaviors, as caregivers with mental health problems typically find it challenging to manage their emotions and behavior in everyday situations, particularly during interactions with their children [16,19]. Secondly, Social Learning Theory highlights that young children develop social skills by seeing and imitating people’s behavior around them in daily life, especially caregivers. When caregivers undergo psychological distress, they may exhibit maladaptive emotional control or diminished social engagement, which children internalize and replicate in their interactions [20]. This maladaptive behavior, observed daily by children and ultimately perceived as “appropriate behavior” in social interactions, can result in adverse socio-emotional development [21]. In conclusion, these frameworks offer a conceptual basis elucidating how caregiver mental health can be associated with social abilities in children over time.

Mental health problems in caregivers are increasingly conceptualized as a multidimensional construct but, in the current study, are categorized into the most common and the most prevalent symptoms: depression, anxiety, and stress [22,23]. Although these dimensions often co-occur [24,25,26], they reflect different emotional, cognitive, and physiological processes that may have unique implications for parenting behaviors and child development [13,27]. Examining each domain independently may provide a more nuanced understanding of how different aspects of caregiver mental health are associated with social outcomes in children.

Guided by the current research gap and theoretical frameworks, in this study we aimed to evaluate how mental health in caregivers is associated with social skill development in preschoolers through a follow-up approach, particularly in urban areas of Indonesia.

## 2. Materials and Methods

### 2.1. Study Design

This follow-up observational study was conducted in an urban area of Indonesia called SJ Ward, B City. All prospective caregivers and teachers were asked to provide consent after being informed about the research, including its objectives, the sequence of research procedures, any potential discomforts that may occur, the rights and responsibilities of participants, and that participation is voluntary and can be withdrawn at any time. Caregivers gave consent for both themselves and their child to participate in the study.

In August 2023 and October 2024, baseline (T1) and follow-up (T2) data were collected. The baseline data (T1) were collected from teachers and caregivers. Caregivers completed the demographic questionnaire and the Depression, Anxiety, Stress Scale (DASS-21), while teachers used SSS-24 to assess social skills in preschoolers. Only teachers participated in the follow-up (T2) data collection procedure to measure the children’s social skills, and all teachers were trained on how to use SSS-24 prior to data collection.

SJ Ward in Bekasi City is a densely populated urban residential district where the study took place. This site was selected because it is located in B City, West Java, the most densely populated province [28], and represents an urban neighborhood near Jakarta, Indonesia’s capital, with a diverse population that may reflect the Indonesian community. Most Indonesian kindergartens operate as private, religion-based institutions, and have a school day from 7.30 to 11.30 [29].

### 2.2. Population and Sample

This investigation targeted all nine SJ kindergartens, and all eligible caregivers and teachers were invited to participate in the study. The inclusion criteria were as follows: (1) caregivers of SJ Ward kindergarteners aged 4–6 who agreed to participate in the study and have their children examined at baseline and follow-up; and (2) teachers who were willing to evaluate children’s social skills by attending researcher training before data collection. The exclusion criteria were as follows: (1) children with special needs; and (2) caregivers currently receiving treatment for health problems (physical and mental).

Twelve of the 383 dyads at SJ Ward Early Childhood Education (PAUD) were excluded from this study owing to special needs. After receiving a detailed explanation about the study, participants agreed to complete the consent form and questionnaire. Due to missing data on demographics, mental health, or children’s social skills, 28 dyads were excluded from the 2023 baseline, and a total of 343 dyads participated in the baseline study.

Teachers evaluated children’s social skills for the 2024 follow-up. Unfortunately, about 41 children were unable to follow-up due to various conditions, and data from 32 children were excluded due to incomplete SSS follow-up, leaving 270 dyads who were included in this study.

### 2.3. Measurements

Mental health problems in caregivers were evaluated by the Indonesian version of DASS-21. DASS-21 is a widely used and validated instrument for assessing depression, anxiety, and stress across diverse populations [24,30,31,32]. The Indonesian version of DASS-21 produced a very good item reliability value of 0.99 and sufficient Pearson reliability (0.89), as well as a Cronbach alpha value, KR-20, of 0.91 [33]. Depression, anxiety, and stress are the three subscales that were included in this self-report questionnaire, and each subscale was analyzed in separate models. This approach was implemented by statistical considerations—intercorrelated between each subscale of DASS—and theoretical perspectives that emphasize these three dimensions have different specific symptoms. Depression is typically characterized by mood disorder and hopelessness, anxiety by heightened feelings of discomfort and fear, and stress by tension and irritability [25], each of which may be differentially related to parenting behaviors and, consequently, children’s social development. Based on the guidelines and previous studies, DASS-21 is used consistently across many iterations and requires participants to assess the frequency of symptoms experienced in the preceding week on a 4-point scale [26,31,33]. Each item was given a rating: 0 = None of the criteria applied to me at all; 1 = Applied to me to a certain extent; 2 = Applied to me to a significant extent; and 3 = Applied to me to a great extent. Higher scores indicated more severe negative mental health issues, whereas lower scores indicated less severe negative mental health issues. The overall score for each subscale was doubled and examined continuously without classifying. In addition, the current study also included the total score of DASS, as it was expected that it would be beneficial to produce a composite measure of negative emotional symptoms and provide a new perspective [26].

Social skills in preschoolers were assessed by kindergarten teachers using the Social Skill Scale (SSS), which comprises 24 items organized into three subscales—(1) assertion, (2) self-control, and (3) cooperation—with each subscale consisting of 8 items. Three responses assess every item—(0) Never, (1) Sometimes, and (2) Always—based on the teacher’s daily observations in kindergartens. The SSS was initially developed and evaluated in a Japanese pediatric population, with a Cronbach’s alpha of 0.91 to 0.93 [34]. This scale has been implemented in numerous countries, including China, Nepal, and Indonesia [35,36,37,38]. The present study employed the validated and reliable Indonesian version of the SSS, exhibiting a Cronbach’s alpha of 0.907 [36]. The final score was calculated by summing the values of all elements within each subdomain, where an elevated score indicates enhanced social abilities.

The current study included covariates: (1) caregivers’ age; (2) caregivers’ educational level; (3) marital status; (4) monthly household income; (5) children’s age; (6) children’s sex; (7) presence of sibling; (8) schools’ accreditation; and (9) children’s social skills in the baseline. The selection of these variables was based on previous research, which mentions demographic factors, family factors, and the school’s environment as determining factors for children’s social skills [3,9,39]. Caregivers answered questions for covariates (1)–(7), and teachers answered covariates (8) and (9).

### 2.4. Statistical Analysis

All data in this study were analyzed using IBM SPSS Statistics version 27.0, with the following steps: descriptive analysis, bivariate correlation analysis, and linear regression mixed-model analysis.

The correlations among the DASS subscales (depression, anxiety, stress) and DASS total score were moderate to strong (r > 0.50, *p* < 0.001), indicating substantial shared variance. Therefore, it would be beneficial to develop a separate model for each subscale to avoid multicollinearity.

Prior to testing the main hypothesis, the nested structure of the participants was examined, as participants came from nine different kindergartens. An unconditional (null) model was employed to calculate the intraclass correlation coefficient (ICC). The ICC for follow-up social skills in children was 0.23, indicating that approximately 23% of the variability among kindergartens can be attributed to differences between them, and multilevel modeling needed to be implemented.

To deal with the hierarchical data structure, we used linear mixed-effects models with random intercepts for kindergarten. This strategy recognizes that observations are not independent and allows intercepts to vary between schools. Only random intercepts were included to ensure model stability, given the few clusters (*n* = 9). Fixed factors included all covariates: caregivers’ age, educational level, marital status, and monthly household income, children’s age and sex, presence of siblings, school accreditation, and children’s social skills at baseline. Continuous predictors were incorporated as covariates, and categorical variables were dummy-coded. We utilized restricted maximum likelihood (REML) to ascertain the parameters, and statistical significance was determined by a *p*-value of less than 0.05.

## 3. Results

### 3.1. Descriptive and Bivariate Correlation Results

Table 1 shows the demographics of the study’s participants, including caregivers and children. The average caregiver age was 34.92 years, with a standard deviation of 5.58, showing that the majority were in their productive adult years, and there were 228 (84.4%) mothers and 42 (15.6%) fathers.

Most caregivers (179, 66.3%) graduated from university (undergraduate). Senior high school accounted for 63 (23.3%) respondents, while junior high and postgraduate education did not account for more than 5%, and only 0.8% attended elementary school. The majority of participants were married and living together (95.8%), only 1.6% were married but living separately for any reason, and the rest (2.6%) were divorced. The income of over half the respondents (52.2%) was above the city’s standard wage; that of 54 (20.0%) was equal to it; and that of 27.8% was below it. This household income reflects urban economic diversity. The average age of preschoolers was 60.00 months, or close to 5 years, with a standard deviation of 7.16 months, and 57.4% were girls. Most participants in the current study also had siblings (84.1%). Finally, 64.8% of schools were B-accredited.

Baseline mental health data for caregivers is shown in Table 2. The total scores for each DASS-21 subscale were multiplied by two and analyzed continuously. The mean stress for caregivers was slightly higher (M = 8.72, SD = 6.19) compared to depression (M = 7.67, SD = 6.65) and anxiety (M = 7.84, SD = 6.04), and the minimum and maximum scores for each subscale were 0 and 42, respectively. Additionally, the mean DASS total score was 24.12 with SD = 15.96, with minimum and maximum scores of 0 and 79, respectively.

Table 3 shows the baseline and follow-up social skills scores of respondents, with an average of 36.06 and a standard deviation of 6.81. The follow-up mean was 40.10 with a standard deviation of 6.17. The maximum SSS score was 48, and the result shows that social skills in children improved after one year.

Table 4 shows that each DASS-21 subscale has significant negative correlations with social skills in children at baseline and follow-up. The results suggest that caregivers with lower mental health issues, such as depression (r = −0.35, *p* < 0.001), anxiety (r = −0.44, *p* < 0.001), stress (r = −0.55, *p* < 0.001), and total DASS score (r = −0.53, *p*< 0.001), exhibited higher development of social skills in children at follow-up.

### 3.2. Mixed-Models Linear Results

As mentioned in the Methods Section, the mixed-model linear analysis was performed with random intercepts for kindergarten because the ICC value was 0.23, indicating significant clustering at the kindergarten level. Thus, all following analyses utilized linear mixed-effects models incorporating random intercepts for kindergarten.

In all models (Table 5), caregivers’ educational level, children’s age, the presence of siblings, and the school’s accreditation were positively associated with higher follow-up social skills scores. In most models, children’s sex was a significant predictor. Lastly, baseline social skills are also considered a significant predictor in the caregiver depression model (see Table 5), suggesting stability in socio-emotional functioning across the one-year period.

Each mixed model developed shows that higher levels of caregivers’ mental health problems—depression, anxiety, stress, and total DASS score—were associated with lower social skills in children at follow-up (see Table 5).

## 4. Discussion

This study’s findings confirm the association of mental health in caregivers at baseline, specifically depression, stress, and anxiety, with the social skills of preschool-aged children in urban Indonesia after one year. The results indicate that higher levels of caregiver depression, anxiety, and stress at baseline were significantly associated with lower levels of social skills in children at follow-up, even after accounting for baseline social skills and relevant covariates. The current study emphasized that baseline social skills were controlled in the analysis; hence, the observed association may reflect differences in developmental trajectories over time rather than solely concurrent relationships between caregiver mental health and child outcomes.

Considered along with previous research, the direction of the associations observed in the present study is comparable with both longitudinal and cross-sectional evidence reported in other settings [9,10,40,41,42,43]. Palmer reported that maternal depression was associated with early child social and emotional development [9]. Another longitudinal study from China also found a significant association between caregiver depression and childhood social development [10]. However, most existing evidence has been derived from high-income countries, while longitudinal data from low- and middle-income countries remain relatively limited. The present findings therefore contribute additional evidence from an urban Indonesian setting, suggesting that similar patterns of association between mental health problems in caregivers and social skills in children may also be observed in different sociocultural contexts. Nevertheless, contextual differences in family structure, cultural norms, and access to resources may shape how these associations emerge, and therefore, the findings should be interpreted within the broader social environment in which families live.

Several theoretical frameworks may help contextualize the observed associations, although we did not directly test the mechanisms proposed by these theories in the present study. The Family Stress Model suggests that psychological distress among caregivers may be linked with changes in family processes, including parenting behaviors and family interactions [44]. Within this framework, caregivers experiencing elevated psychological distress may demonstrate different patterns of responsiveness, emotional availability, or consistency in interactions with children [45]. These interaction patterns have been discussed in prior research as a factor associated with social skills development in children [21,46]. In addition, Social Learning Theory proposes that children acquire social and emotional behaviors partly through observing and imitating caregivers [20]. When caregivers undergo mental health problems, they may struggle to display affection or emotion, causing them to exhibit maladaptive emotional reactions, which children may absorb and replicate in their daily social interactions [10,16,19,21]. However, because parenting practices and observational learning processes were not measured in the present study, these theoretical perspectives should be interpreted as possible explanatory frameworks rather than empirically tested mechanisms.

In the current study, we also highlighted a substantial clustering effect at the kindergarten level, with approximately 23% of the variance in social skills in children attributable to differences among kindergartens, suggesting that children attending the same kindergarten tended to show more similar social skill outcomes than children attending different schools. In the current study, we only included schools’ accreditation as a variable, limiting the interpretation of variations in schools that might comprise school quality, teacher–child interactions, school environment, peer relationships, and the curriculum related to social–emotional learning activities, which may be associated with outcomes in children [47]. Because these contextual factors were not directly measured in the present study, they should be interpreted as possible explanations rather than empirically tested mechanisms. Future research incorporating school-level variables could help clarify the role of educational environments in social development in children.

In addition, the current study not only includes the mental health of caregivers but also several covariates of demographic factors of caregivers and children, which have been identified as significant factors in previous studies [3,9,43]. Children’s age and sex have been discussed as determining factors for their social skills, as children develop their social skills as they grow, and girls are often mentioned to develop their skills earlier than boys [9,48]. Caregiver education has also been frequently associated with developmental outcomes in children, possibly reflecting differences in access to information, parenting knowledge, and opportunities to provide stimulating environments [49]. Lastly, the presence of siblings has been discussed as a factor related to social experiences in children, as interactions between siblings may provide opportunities for practicing cooperation, negotiation, and conflict resolution [50]. Although these factors were included as covariates rather than primary variables of interest, their inclusion helps situate the association between caregiver mental health and children’s social skills within a broader family and developmental context.

Several alternative explanations should also be considered when interpreting the findings. First, unmeasured aspects of the caregiving environment, such as parenting style, family conflict, or levels of social support, may be associated with both caregiver mental health and children’s social development. Second, environmental stressors common in urban settings, including economic pressures or limited access to childcare and support services, may be related to both caregiver psychological well-being and children’s developmental experiences. Third, the possibility of bidirectional associations cannot be completely ruled out. For instance, children with more challenging behavioral characteristics may also be associated with increased caregiver stress over time, although baseline social skills were statistically controlled in the present study. These considerations highlight the complexity of family processes and suggest that caregiver mental health should be interpreted as one factor within a broader ecological system related to social development in children.

Finally, this study adds to the scarce follow-up evidence from low- and middle-income countries (LMICs), specifically within urban Indonesian contexts. Caregivers residing in urban environments often initially exhibit stress symptoms that can subsequently progress to clinical depression in the absence of suitable intervention. The persistent doubt and stigma around mental health issues, which continue to be regarded as taboo, frequently intensify depression [51]. These findings highlight the importance of improving awareness of and accessibility to mental health services. These circumstances may exacerbate the relationship between caregiver mental health and parenting practices and, subsequently, child development outcomes [14,51]. Consequently, understanding caregiver mental health within its sociocultural and structural context is crucial for formulating effective therapies.

The findings also have important practical implications, as this is the first investigation to clarify the association between mental health problems in caregivers and social skills in children, using a follow-up approach in an urban Indonesian context. Because this study is observational, the findings should be interpreted as associations rather than causal relationships. First, integrating mental health screenings by utilizing a valid checklist (i.e., DASS-21) for caregivers into early childhood education and community health services could enable the early identification of at-risk families. In Indonesian cultures and other LMICs, mental health problems are often stigmatized and considered taboo to discuss, which may discourage caregivers from seeking professional help, leading to unrecognized and untreated mental health problems. Therefore, including the community would be significant. Second, the findings underscore the relationship between caregiver psychological well-being and children’s social development. Although causal relationships cannot be inferred, the observed associations suggest that caregiver mental health may represent an important contextual factor to consider in family-oriented and early childhood support initiatives. Strengthening awareness of and accessibility to caregiver mental health services may therefore contribute to more comprehensive approaches to promoting child well-being, particularly in urban LMIC settings.

Notwithstanding these achievements, several limitations must be acknowledged. Mental health in caregivers was assessed by a self-report instrument, while social skills in children were evaluated solely through teacher reports. This single-informant approach for each variable may introduce informant-related bias. Caregivers may under- or overreport their mental health status due to social desirability or stigma, particularly within the Indonesian cultural context. Similarly, teacher assessments of social skills in children may be influenced by subjective perceptions or classroom-specific dynamics despite advanced training. A future study should utilize multi-informant data (e.g., parent–teacher combined reports) to improve the robustness of the measurements. In addition, this study was conducted in a single urban area and included a relatively small number of kindergartens, all of which were private institutions. This context-specific sampling may limit the generalizability of the findings to other populations, particularly rural areas or public early childhood education settings, where socio-economic conditions, caregiving practices, and access to resources may differ substantially. Therefore, caution is warranted when extending these findings beyond similar urban Indonesian contexts.

This study also experienced a notable dropout rate between baseline and follow-up, which may introduce attrition bias. Participants who were lost to follow-up may differ systematically from those who remained in the study, potentially influencing the observed relationships. Finally, although several relevant covariates were included, we did not account for other potentially important factors in this study, such as parenting style, home-rearing environment, caregivers’ mental health at T2, social support systems, and school environment. These unmeasured variables may play a mediating or moderating role in the relationship between caregiver mental health and child social development. Future research incorporating more comprehensive and multi-method assessments is recommended to further elucidate these pathways.

## Figures and Tables

**Table 1:** Demographics of participants (N = 270).

Variables	Categories	*n*	%
Caregivers’ Age (Years)	Mean + SD: 34.92 + 5.58		
Relationship	Father	42	15.6
	Mother	228	84.4
Caregivers’ Education	Elementary	2	0.8
	Junior High School	13	4.8
	Senior High School	63	23.3
	Undergraduate	179	66.3
	Postgraduate	13	4.8
Caregivers’ Marital Status	Married—Living Together	259	96.0
	Married—Living Separately	4	1.4
	Divorce	7	2.6
Household Income ^1^	<City Standard	75	27.8
	=City Standard	54	20.0
	>City Standard	141	52.2
Child’s Age (Months)	Mean + SD: 60.00 + 7.16		
Child’s Sex	Boy	115	42.6
	Girl	155	57.4
Presence of Sibling	Yes	227	84.1
	No	43	15.9
School’s Accreditation	A	78	28.9
	B	175	64.8
	C	17	6.3

^1^ City standard salary at baseline was approximately USD 340.

**Table 2:** Descriptive results of caregivers’ mental health (N = 270).

Variable	Mean	SD	Min	Max
Depression ^1^	7.67	6.65	0	42
Anxiety ^1^	7.84	6.04	0	42
Stress ^1^	8.72	6.19	0	42
DASS Total Score ^2^	24.12	15.96	0	79

^1^ DASS subscale range scores: 0–42; ^2^ DASS total score range scores: 0–126.

**Table 3:** Descriptive results of children’s social skills at baseline (T1) and follow-up (T2) (N = 270).

Variable	Mean	SD	Min	Max
Children’s Social Skills T1 ^1^	36.06	6.81	3	46
Children’s Social Skills T2 ^1^	40.10	6.17	20	48

^1^ SSS range scores: 0–48.

**Table 4:** Bivariate correlation results of caregivers’ mental health and children’s social skills (N = 270).

Variable	1	2	3	4	5	6
1. Caregivers’ Depression	1.00					
2. Caregivers’ Anxiety	0.69 **	1.00				
3. Caregivers’ Stress	0.58 **	0.67 **	1.00			
4. Caregivers’ Total DASS score	0.86 **	0.89 **	0.85 **	1.00		
5. Children’s Social Skills T1	−0.19 **	−0.22 **	−0.22 **	−0.25 **	1.00	
6. Children’s Social Skills T2	−0.35 **	−0.44 **	−0.55 **	−0.53 **	0.31 **	1.00

** *p* < 0.01.

**Table 5:** Mixed-effects models examining associations between caregivers’ baseline mental health and children’s social skills at one-year follow-up (N = 270).

Predictor	Model 1 Covariates	Model 2 Depression	Model 3 Anxiety	Model 4 Stress	Model 5 Total DASS Score
Intercept	−7.27 (3.19) * [−13.57, −0.97]	−4.24 (3.23) [−10.62, 2.15]	−3.59 (3.23) [−9.97, 2.80]	0.57 (3.22) [−5.79, 6.93]	0.84 (3.28) [−5.64, 7.32]
Caregivers’ Age	−0.01 (0.04) [−0.09, 0.06]	−0.01 (0.04) [−0.08, 0.06]	−0.01 (0.03) [−0.08, 0.06]	−0.01 (0.03) [−0.08, 0.06]	−0.01 (0.03) [−0.08, 0.05]
Caregivers’ Education	1.03 (0.33) ** [0.38, 1.68]	0.87 (0.32) ** [0.24, 1.51]	0.85 (0.31) ** [0.23, 1.47]	0.67 (0.31) * [0.07, 1.27]	0.59 (0.30) * [0.00, 1.18]
Marital Status	0.48 (0.61) [−0.73, 1.69]	0.42 (0.59) [−0.75, 1.60]	0.49 (0.58) [−0.65, 1.63]	0.41 (0.56) [−0.69, 1.52]	0.44 (0.55) [−0.64, 1.52]
Household Income	−0.28 (0.25) [−0.77, 0.20]	−0.29 (0.24) [−0.77, 0.18]	−0.28 (0.23) [−0.74, 0.18]	−0.26 (0.23) [−0.71, 0.18]	−0.28 (0.22) [−0.72, 0.15]
Child’s Age	0.53 (0.03) *** [0.46, 0.59]	0.50 (0.03) *** [0.43, 0.56]	0.47 (0.03) *** [0.41, 0.53]	0.44 (0.03) *** [0.37, 0.50]	0.42 (0.03) *** [0.36, 0.48]
Child’s Sex	0.84 (0.42) * [0.00, 1.68]	0.79 (0.41) [−0.02, 1.60]	0.88 (0.40) * [0.09, 1.67]	0.81 (0.39) * [0.05, 1.57]	0.79 (0.38) * [0.04, 1.54]
Presence of Siblings	2.80 (0.59) *** [1.63, 3.96]	2.76 (0.57) *** [1.63, 3.89]	2.56 (0.56) *** [1.46, 3.66]	2.85 (0.54) *** [1.78, 3.91]	2.65 (0.53) *** [1.61, 3.69]
Schools’ Accreditation	2.67 (0.88) ** [0.86, 4.48]	3.10 (0.89) ** [1.28, 4.91]	4.04 (0.93) *** [2.16, 5.92]	3.63 (0.88) *** [1.86, 5.40]	4.70 (0.93) *** [2.85, 6.55]
Children’s Social Skills T1	0.08 (0.03) * [0.01, 0.15]	0.07 (0.03) * [0.01, 0.14]	0.06 (0.03) [−0.01, 0.12]	0.05 (0.03) [−0.01, 0.12]	0.05 (0.03) [−0.02, 0.11]
Caregivers’ Depression		−0.15 (0.04) *** [−0.23, −0.08]			
Caregivers’ Anxiety			−0.22 (0.04) *** [−0.30, −0.14]		
Caregivers’ Stress				−0.27 (0.04) *** [−0.34, −0.19]	
Caregivers’ DASS Total Score					−0.13 (0.02) *** [−0.16, −0.10]

Note: Values are B (SE); 95% confidence intervals are shown in brackets. A random intercept for kindergarten was included in all models. * *p* < 0.05, ** *p* < 0.01, and *** *p* < 0.001. Reference categories: divorced (marital status), household income below the city standard, boy (child sex), no siblings, school accreditation C, and elementary education.

## Data Availability

Data are not publicly available due to privacy or ethical restrictions.

## Abbreviations

The following abbreviations are used in this manuscript:

DASS	Depression Anxiety Stress Scale
SSS	Social Skills Scale
